# The pH Response and Sensing Mechanism of n-Type ZnO/Electrolyte Interfaces

**DOI:** 10.3390/s90907445

**Published:** 2009-09-16

**Authors:** Safaa Al-Hilli, Magnus Willander

**Affiliations:** Department of Science and Technology, Campus Norrköping, Linköping University, SE-60174 Norrköping, Sweden; E-Mail: Magnus.Willander@itn.liu.se

**Keywords:** ZnO nanorods, pH sensor, electrolyte double layer, n-type ZnO-electrolyte interface, fermi level pinning, potentiometric measurements

## Abstract

Ever since the discovery of the pH-sensing properties of ZnO crystals, researchers have been exploring their potential in electrochemical applications. The recent expansion and availability of chemical modification methods has made it possible to generate a new class of electrochemically active ZnO nanorods. This reduction in size of ZnO (to a nanocrystalline form) using new growth techniques is essentially an example of the nanotechnology fabrication principle. The availability of these ZnO nanorods opens up an entire new and exciting research direction in the field of electrochemical sensing. This review covers the latest advances and mechanism of pH-sensing using ZnO nanorods, with an emphasis on the nano-interface mechanism. We discuss methods for calculating the effect of surface states on pH-sensing at a ZnO/electrolyte interface. All of these current research topics aim to explain the mechanism of pH-sensing using a ZnO bulk- or nano-scale single crystal. An important goal of these investigations is the translation of these nanotechnology-modified nanorods into potential novel applications.

## 1. Introduction

In the study of electrochemistry the electrode was used for a long time only as a source, or a sink, of electrons provided by an electronic conductor with low resistivity. This paradigm has changed, largely due to the interest shown by electrochemists in the field of metal oxide semiconductors.

In contrast to metal electrodes, metal oxide semiconductor electrodes are well-suited to address some of the fundamental predictions of interfacial electron transfer theories. An ideal semiconductor has no electronic levels in the band gap region; therefore, for an n-type material, only electrons with energies near the conduction band can contribute to the cathodic interfacial current flow.

Unlike in a metal electrode, the driving force at a semiconductor electrode cannot be changed by varying the potential of the electrode. This situation occurs because the differential capacitance of a non-degenerately doped semiconductor electrode is much smaller than the differential capacitance of the electrolyte. Essentially all of the applied potential drops across the electrode and not across the electrolyte. Hence, the only methods of changing the driving force are to vary the energetics of the redox species in the solution, or to vary the identity or chemical state of the semiconductor surface.

ZnO is a material of interest in fundamental studies of the semiconductor/electrolyte (SC/EL) interface. ZnO also has practical applications in transparent conducting electrodes, sensors, and topical medical pastes that take advantage of its UV-induced generation of peroxide species, which act as sterilizing agents. ZnO nanorods can act as charge collecting electrodes in dye-sensitized photo-electrochemical cells. Many of these applications are based on electrochemical reactions at the ZnO surface, which involve free carriers.

Semiconductor problems have also stimulated many physicists to probe electrochemical questions. In this paper, we discuss several areas in which ZnO semiconductors have offered new perspectives on electrochemistry. For this discussion, we have chosen the principal areas listed below.

1. Energy levels in semiconductors and electrolytes
2. The electrical double layer
3. Mapping of the semiconductor band edge positions relative to solution redox levels (pH-sensing).
4. The role of surface states.

We will, in the present paper, look at the distributions of charge, potential, and capacitance at the zinc oxide-electrolyte interface. The distinction between metal and semiconductor electrodes is important when we consider the electrostatics across the corresponding solid-liquid interfaces.

## 2. Energy Levels in Semiconductors and Liquids

### 2.1. Electron Energy Levels in Semiconductors and the Energy Band Model

**Figure 1.**
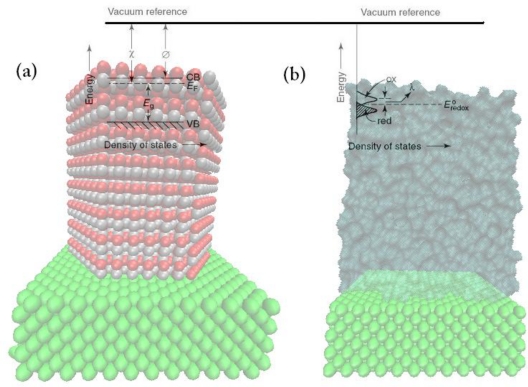
Energy levels in (a) a semiconductor and (b) a redox electrolyte, shown with a common vacuum reference scale, where χ and φ are the semiconductor electron affinity and work function, respectively.

The quantum theory of solids presents a complete description of the energy levels in a semiconductor, the nature of charge carriers, and laws governing their motion [1,2]. The filled energy states correspond to the valence band (its upper edge is denoted as *E*_v_) and the empty states to the conduction band (its lower edge is denoted *E*_c_). The energy bands are separated by the band gap, *E*_g_, as illustrated in Figure 1a. In solid state physics, the vacuum level is taken as the zero energy reference.

The density of energy states within the energy bands increases with the square root of energy above the conduction band or below the valence band edge and is given by:

(1) $$N_{C}=\frac{8\sqrt{2\pi }}{h^{3}}{\left(m_{e}^{*}\right)}^{\frac{3}{2}}{\left(E-E_{c}\right)}^{\frac{1}{2}}$$

for the conduction band and:

(2) $$N_{C}=\frac{8\sqrt{2\pi }}{h^{3}}{\left(m_{h}^{*}\right)}^{\frac{3}{2}}{\left(E-E_{V}\right)}^{\frac{1}{2}}$$

for the valence band, in which *h* is Planck’s constant and *m*_e_* and *m*_h_* are the effective masses of electrons and holes, respectively. The electron and hole densities in the conduction and valence bands, respectively, are related to the corresponding Fermi levels, *E_F,n_* and *E_F,p_*, by:

(3) $$n=N_{c}\;\;\text{exp}\left(-\frac{E_{c}-E_{F,n}}{\mathrm{kT}}\right)$$

(4) $$p=N_{V}\;\;\text{exp}\left(-\frac{E_{V}-E_{F,p}}{\mathrm{kT}}\right)$$

in which *N*_c_ and *N*_v_ are given by Equations (1) and (2). At equilibrium, the Fermi levels of electrons and holes are identical, i.e., *E_F,n_* = *E_F,p_* = *E*_F_, *n* = *n*_0_, and *p* = *p*_0_. Inserting these values into Equations (3) and (4) and multiplying these two Equations, one obtains, at equilibrium:

(5) $$n_{i}^{2}=n_{o}\;p_{o}=N_{C}\;N_{V}\;\;\text{exp}\left(\frac{E_{C}-E_{V}}{\mathrm{kT}}\right)=N_{C}\;N_{V}\;\;\text{exp}\left(-\frac{E_{g}}{\mathrm{kT}}\right)$$

where *n_i_*^2^ is a material constant which decreases exponentially with increasing band gap, E_g_. The relative position of the Fermi level, *E*_F_, depends on the electron and hole concentration, i.e., on the doping of the semiconductor. The equilibrium carrier densities in the conduction and valence bands, *n*_0_ and *p*_0_, can be calculated using Equations (3) and (4). Typical carrier densities in semiconductors range from 10^15^ to 10^19^ cm^−3^. This level corresponds to a range of Fermi levels, *E*_F_, of 0.04–0.25 eV with respect to one of the energy bands. Thus, only a small portion of the energy states at the edges are occupied.

### 2.2. Solution-Redox Levels

Considerations of interfacial electron transfer require knowledge of the relative positions of the participating energy levels in the two phases (semiconductor and solution). Besides the Fermi level of the redox system, this model introduces the existence of occupied and empty energy states corresponding, respectively, to the reduced and oxidized species of the redox system. The model leads to a Gaussian distribution of redox states versus electron energy, as illustrated in the Figure 1b. The distribution functions for the states are given by [3]:

(6) $$D_{\mathrm{ox}}=\text{exp}\left[-\frac{{\left(E-E_{F,\mathrm{redox}}-\lambda \right)}^{2}}{4\mathrm{kT}\lambda }\right]$$

(7) $$D_{\mathrm{red}}=\text{exp}\left[-\frac{{\left(E-E_{F,\mathrm{redox}}+\lambda \right)}^{2}}{4\mathrm{kT}\lambda }\right]$$

in which λ is the well-known reorganization energy of electron transfer theory [4]. Generally, λ falls in the range of 0.5–2 eV, depending on the interaction of the redox molecule with the solvent. The Gaussian type of distribution is a consequence of the assumption that the fluctuation of the solvation shell corresponds to a harmonic oscillation. Models for redox energy levels in solution have been exhaustively treated in several articles [3,5–10].

### 2.3. n-Type Semiconductor-Electrolyte Systems at Equilibrium

It should be emphasized that the Fermi level is actually the electrochemical potential of electrons in the solid. The electrochemical potential of electrons in a redox electrolyte is given by the Nernst expression:

(8) $$E_{\mathrm{redox}}=E_{\mathrm{redox}}^{o}+\frac{\mathrm{RT}}{\mathrm{nF}}\;\;\text{ln}\;\left\lfloor \frac{c_{\mathrm{ox}}}{c_{\mathrm{red}}}\right\rfloor$$

or:

(9) $${\bar{\mu }}_{e,\mathrm{redox}}=\mu_{\mathrm{redox}}^{o}+\mathrm{kT}\;\;\text{ln}\;\left(\frac{c_{\mathrm{ox}}}{c_{\mathrm{red}}}\right)$$

in which *c*_ox_ and *c*_red_ are the concentrations (roughly equal to the activities) of the oxidized and reduced species of the redox couple system, respectively. The parameter *E_redox_* = *μ_e,redox_* can be equated to the Fermi level *E_F,redox_* in the electrolyte. In this case, the electrochemical potential of electrons in a redox system is equivalent to the Fermi level, *E_F,redox_*; i.e.,:

(10) $$E_{F,\mathrm{redox}}={\bar{\mu }}_{e,\mathrm{redox}}$$

on the absolute scale [9]. The task now is to relate the electron energy levels in the solid and liquid phases on a common basis.

In semiconductor solid-state physics, the vacuum level has been adopted as the standard reference. In contrast, electrochemists express redox potentials on a conventional scale, using the normal hydrogen electrode (NHE) or the saturated calomel electrode (SCE) as a reference point. The electrochemical potential of a redox system is usually given with respect to the normal hydrogen electrode (NHE). Using an absolute energy scale, the energy of a redox couple is given by [11]:

(11) $$E_{E,\mathrm{redox}}=E_{\mathrm{ref}}-{\mathrm{qV}}_{\mathrm{redox}}$$

in which V_redox_ is the redox potential vs NHE, and *E*_ref_ is the energy of the reference electrode versus the vacuum level. The determination of *E*_ref_ has been the subject of several calculations [12,13]. The values derived by various authors range from 4.3 to 4.7 eV. Usually, an average value of *E*_ref_ = 4.5 eV for NHE is used, so that Equation 11 yields:

(12) $$E_{F,\mathrm{redox}}=-4.5-{\mathrm{qV}}_{\mathrm{redox}}$$

with respect to the vacuum level. The relationship between the various energy scales for the solid and liquid phases is shown in Figure 1b.

When a semiconductor is immersed in a redox electrolyte, the electrochemical potential is disparate across the interface. In order for the two phases to be in equilibrium, their electrochemical potential must be the same. The electrochemical potential of the solution is determined by the redox potential of the electrolyte species, and the redox potential of the semiconductor is determined by its Fermi level. A movement of charge between the semiconductor and the solution is required to equilibrate the two phases if their redox potentials (Fermi levels) do not lie at the same energy. The excess charge that is now located on the semiconductor does not lie at the surface, as it would for a metallic electrode but extends into the electrode for a significant distance. This region is referred to as the space charge region and has an associated electric field.

**Figure 2.**
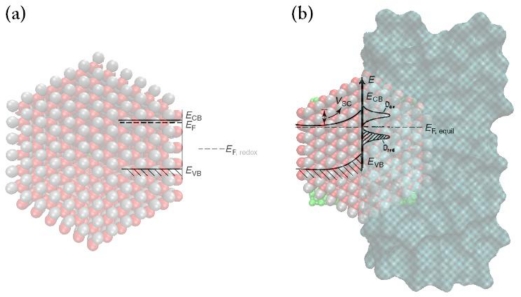
Energy levels of n-type ZnO before equilibrium (a) and the band bending in n-type ZnO electrode upon equilibration of the Fermi level of the semiconductor with the redox species (b).

For an n-type semiconductor electrode at open circuit, the Fermi level is typically higher than the redox potential of the electrolyte. Electrons will therefore be transferred from the electrode into the solution. There is thus a positive charge associated with the space charge region, and this is reflected in an upward bending of the band edges by an energy of V_sc_, which depends on the doping (see Figure 2). Since the majority charge carrier of the semiconductor has been removed from this region, this region is also referred as the depletion layer. After contact, the Fermi levels of the semiconductor and the redox system must be equal on both sides of the interface [14]:

(13) $$E_{F}=E_{F,\mathrm{redox}}$$

and a built in-voltage, V_sc_, develops within the semiconductor phase.

## 3. The Electrical Double Layer

As is the case for metals, the Helmholtz layer of a semiconductor is developed by adsorption of ions or molecules on the material surface, by oriented dipoles, or, especially in the case of oxides, by the formation of surface bonds between the solid surface and the species in solution. For amphoteric ZnO surfaces, we can write local equilibrium reactions and equilibrium constants [15–17]:

(14) $$\equiv \mathrm{ZnOH}+H_{\left(\mathrm{aq}\right)}^{+}=\;\equiv {\mathrm{ZnOH}}_{2}^{+}$$

(15) $$\equiv {\mathrm{ZnO}}^{-}+H_{\left(\mathrm{aq}\right)}^{+}=\;\equiv \mathrm{ZnOH}$$

K_1_ and K_2_ are estimated from known thermodynamic data for equilibria of the reactions:

(16) $${\mathrm{Zn}(\mathrm{OH})}_{2(S)}+H_{\left(\mathrm{aq}\right)}^{+}={\mathrm{ZnOH}}_{\left(\mathrm{aq}\right)}^{+}+H_{2}O$$

(17) $${\mathrm{ZnO}}_{2}H_{\left(\mathrm{aq}\right)}^{+}+H_{\left(\mathrm{aq}\right)}^{+}={\mathrm{Zn}(\mathrm{OH})}_{2(S)}$$

(18) $$K_{1}=\frac{\left[{\mathrm{ZnOH}}_{2}^{+}\right]}{\left[\mathrm{ZnOH}\right]\;\left[H_{\mathrm{aq}}^{+}\right]}$$

(19) $$K_{2}=\frac{\left[\mathrm{ZnOH}\right]}{\left[{\mathrm{ZnO}}^{-}\right]\;\left[H_{\mathrm{aq}}^{+}\right]}$$

the equilibrium conditions of reactions 14 and 15 require that:

(20) $${\bar{\mu }}_{\mathrm{ZnOH}}+{\bar{\mu }}_{H_{S}^{+}}={\bar{\mu }}_{{\mathrm{ZnOH}}_{2}^{+}}$$

(21) $${\bar{\mu }}_{{\mathrm{ZnO}}^{-}}+{\bar{\mu }}_{H_{S}^{+}}={\bar{\mu }}_{\mathrm{ZnOH}}$$

The concentration of protons at some location near the solid surface in the double layer is related to the bulk concentration by the Boltzmann distribution:

(22) $$\left[H_{S}^{+}\right]=\left[H_{b}^{+}\right]\;\;\text{exp}\left(-\frac{e\psi_{o}}{\mathrm{kT}}\right)$$

Where *ψ_0_* is the surface potential of the solid.

Thus, at the surface:

(23) $$K_{1}=\frac{\left[{\mathrm{ZnOH}}_{2}^{+}\right]}{\left[\mathrm{ZnOH}\right]\;\left[H_{\mathrm{aq}}^{+}\right]}\;\text{exp}\;\left(-\frac{e\psi_{o}}{\mathrm{kT}}\right)$$

(24) $$K_{2}=\frac{\left[\mathrm{ZnOH}\right]}{\left[{\mathrm{ZnO}}^{-}\right]\;\left[H_{\mathrm{aq}}^{+}\right]}\;\text{exp}\;\left(-\frac{e\psi_{o}}{\mathrm{kT}}\right)$$

In addition to these reactions involving protons or hydroxyl ions, electrolyte counterions could adsorb to neutralize the surface charge (since the diffuse layer charge may be significantly less than the surface charge). To account for specific adsorption of electrolyte ions, Yates *et al.* [18] proposed the formation of ion pairs or surface complexes at charged surfaces, e.g.:

(25) $$\equiv {\mathrm{ZnO}}^{-}+{\mathrm{Na}}_{S}^{+}=\;\equiv {\mathrm{ZnO}}^{-}{\mathrm{Na}}^{+}$$

(26) $$\equiv {\mathrm{ZnOH}}_{2}^{+}+{\mathrm{Cl}}_{S}^{-}=\;\equiv {\mathrm{ZnOH}}_{2}^{+}{\mathrm{Cl}}^{-}$$

The concentrations of ions in the electrical double layer are expressed in terms of the Boltzmann distribution, e.g.:

(27) $${\left[{\mathrm{Na}}^{+}\right]}_{i}=\left[{\mathrm{Na}}^{+}\right]\text{exp}\left(-\frac{e\psi_{\beta }}{\mathrm{kT}}\right)$$

(28) $${\left[{\mathrm{Cl}}^{-}\right]}_{i}=\left[{\mathrm{Cl}}^{-}\right]\text{exp}\left(-\frac{e\psi_{\beta }}{\mathrm{kT}}\right)$$

where the dissociation constants of reactions (25) and (26) are:

(29) $$K_{{\mathrm{Na}}^{+}}=\frac{\left[{\mathrm{ZnO}}^{-}{\mathrm{Na}}^{+}\right]}{\left[{\mathrm{ZnO}}^{-}\right]\;\left[{\mathrm{Na}}^{+}\right]}\;\;\;\;\text{exp}\left(-\frac{e\psi_{\beta }}{\mathrm{kT}}\right)$$

(30) $$K_{{\mathrm{Cl}}^{-}}=\frac{\left[{\mathrm{ZnOH}}_{2}^{+}{\mathrm{Cl}}^{-}\right]}{\left[{\mathrm{ZnOH}}_{2}^{+}\right]\;\left[{\mathrm{Cl}}^{-}\right]}\;\;\;\;\text{exp}\left(-\frac{e\psi_{\beta }}{\mathrm{kT}}\right)$$

where *ψ_β_* is the mean potential at the plane of specifically adsorbed counterions. We can write the reactions as complex ionizations:

(31) $$\equiv \mathrm{ZnOH}+{\mathrm{Na}}^{+}=\;\equiv {\mathrm{ZnO}}^{-}{\mathrm{Na}}^{+}+H_{S}^{+}$$

(32) $$\equiv {\mathrm{ZnOH}}_{2}^{+}{\mathrm{Cl}}^{-}=\;\equiv \mathrm{ZnOH}+H_{S}^{+}+{\mathrm{Cl}}^{-}$$

where the dissociation constants of reactions (31) and (32) are:

(33) $${}^{*}K_{{\mathrm{Na}}^{+}}=K_{2}\cdot K_{{\mathrm{Na}}^{+}}$$

(34) $${}^{*}K_{{\mathrm{Cl}}^{-}}=\frac{K_{1}}{K_{{\mathrm{Cl}}^{-}}}$$

and:

(35) $${}^{*}K_{{\mathrm{Na}}^{+}}=\frac{\left[{\mathrm{ZnO}}^{-}{\mathrm{Na}}^{+}\right]\;\left[H_{S}^{+}\right]}{\left[\mathrm{ZnOH}\right]\;\left[{\mathrm{Na}}^{+}\right]}\;\text{exp}\;\left(\frac{e}{\mathrm{kT}}\left(\psi_{o}-\psi_{\beta }\right)\right)$$

(36) $${}^{*}K_{{\mathrm{Cl}}^{-}}=\frac{\left[\mathrm{ZnOH}\right]\;\left[H_{S}^{+}\right]\;\left[{\mathrm{Cl}}^{-}\right]}{\left[{\mathrm{ZnOH}}_{2}^{+}\;{\mathrm{Cl}}^{-}\right]}\;\text{exp}\;\left(\frac{e}{\mathrm{kT}}\left(\psi_{\beta }-\psi_{d}\right)\right)$$

The surface charge, *σ_0_*, is given by:

(37) $$\sigma_{o}=B\left(\left[{\mathrm{ZnOH}}_{2}^{+}\right]+\left[{\mathrm{ZnOH}}_{2}^{+}\;{\mathrm{Cl}}^{-}\right]-\left[{\mathrm{ZnO}}^{-}\right]-\left[{\mathrm{ZnO}}^{-}{\mathrm{Na}}^{+}\right]\right)$$

The charge in the mean plane of specifically adsorbed counterions is:

(38) $$\sigma_{\beta }=B\left(\left[{\mathrm{ZnO}}^{-}{\mathrm{Na}}^{+}\right]-\left[{\mathrm{ZnOH}}_{2}^{+}{\mathrm{Cl}}^{-}\right]\right)$$

(39) $$B=10^{6}\;F/A$$

where:

B = a conversion factor from moles/liter to μC/cm^2^ of charge.
A = the surface area of ZnO immersed in the solution (cm^3^/liter)
F = the Faraday constant
[Concentration] = moles/liter

and the charge in the diffusion layer is:

(40) $$\sigma_{d}=-11.74C^{0.5}\text{sinh}\left(\frac{\mathrm{ze}\psi_{d}}{2\mathrm{kT}}\right)$$

where:

C = the bulk concentration
Z = the charge of the supporting electrolyte counter ion in the diffusion layer.
*ψ_d_* = the mean potential at the start of the diffusion layer.

Electroneutrality requires that:

(41) $$\sigma_{o}+\sigma_{\beta }+\sigma_{d}=0$$

Where *σ_0_*, *σ_d_* and *σ_0_* in μC/cm^2^.

Ionized surface sites are confined to a mean surface plane, and specifically adsorbed counterions are confined to a second mean potential plane at a distance β.

Constant capacitances are assumed in the regions between the planes, yielding charge potential relationships [19–21]:

(42) $$C_{1}=\frac{\sigma_{o}}{\psi_{o}-\psi_{\beta }}=C_{H}$$

(43) $$C_{2}=-\frac{\sigma_{d}}{\psi_{\beta }-\psi_{d}}=C_{G}$$

where C_H_ and C_G_ are, respectively, the Helmholtz and Gouy integral capacitances of the inner regions.

The surface species are distributed among the total number of sites available (surface mass balance), N_s_ (in μC/cm^2^), which is given by:

(44) $$N_{S}=B\left(\left[{\mathrm{ZnOH}}_{2}^{+}\right]+\left[{\mathrm{ZnOH}}_{2}^{+}\;{\mathrm{Cl}}^{-}\right]+\left[\mathrm{ZnOH}\right]+\left[{\mathrm{ZnO}}^{-}\right]+\left[{\mathrm{ZnO}}^{-}{\mathrm{Na}}^{+}\right]\right)$$

If more than one surface layer participates in this exchange process, the effect of the slow process becomes proportionately larger, but since the ions must penetrate more deeply into the solid, the rate of the exchange reaction will decrease with time. Furthermore, the exchange between surface hydroxyls and anions in solution is accompanied by consumption or release of OH^−^ ions, which alters the pH of the solution in such a way that the driving force for the exchange reaction is diminished. With time, therefore, the observed pH drift should decrease and cease when a stationary state has been reached.

## 4. n-Type Semiconductor-Electrolyte Interface: Physical

The distribution of electric charge at the interface between an n-type semiconductor electrode and an electrolyte solution can be obtained by measuring the interfacial capacitance. There are three regions which are distinguished in the model [22]:

1. The space charge region in the semiconductor, bounded on one side by the surface of the semiconductor and decaying into the electrode bulk. The length of the space charge region depends on the doping density. There is a characteristic region inside the semiconductor within which the charge would have been removed by the equilibration process. Beyond this boundary, the ionized donors (for an n-type semiconductor), have their compensating charge (electrons), and the semiconductor as a whole is electrically neutral. This layer is the space charge region, also known as the depletion layer, so termed because the layer is depleted of the majority carrier.
2. The Helmholtz region. This region adjacent to the semiconductor surface is measured from the plane through the center of the surface atoms of the semiconductor to the Helmholtz plane (the plane through the center of the ions of the electrolyte at their point of closest approach to the semiconductor surface) and is typically 0.3–0.6 nm in thickness.
3. The space charge region in the electrolyte, bounded on one side by the outer Helmholtz plane and decaying into the bulk of the electrolyte. This region is also called the Gouy diffuse layer.

## 5. n-Type Semiconductor-Electrolyte Interfaces (without Surface States)

For this case, the total excess charge per unit area in the semiconductor space charge region is equal and opposite to the excess charge in the electrolyte (assuming the effect of the diffuse double layer in the electrolyte can be eliminated by using electrolytes of sufficiently high concentration, ∼1 M).

The electric charge needed for Fermi level equilibrium in the semiconductor phase originates from the donor impurities (rather than from bonding electrons in the semiconductor lattice). Thus, the depletion layer that arises as a consequence within the semiconductor contains positive charges from these ionized donors. The Fermi level in the semiconductor, E_F,n_, moves down. This process stops when the Fermi level is the same on either side of the interface. The rather substantial difference in the density of states on either side dictates that E_F,n_ moves further than the corresponding level, E_F,redox_, in the electrolyte [23].

**Figure 3.**
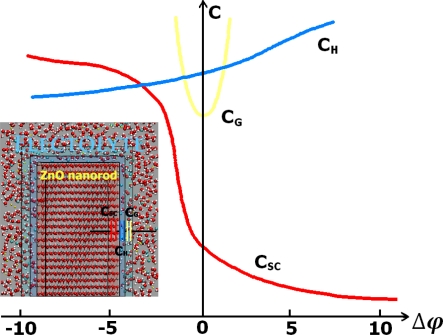
Potential dependence of the capacitance components of the n-type semiconductor-electrolyte interface.

The band-bending phenomenon is by no means unique to the semiconductor-electrolyte interface. Analogous electrostatic adjustments occur whenever two dissimilar phases are in contact. An important point of distinction from the corresponding situation involving a metal is that the charge, and thus the associated potential drop, is concentrated at the surface, penetrating at most a few angstroms into the interior. Stated differently, the high electrical conductivity of a metal cannot support an internal electric field. Thus, when a metal electrode comes into contact with an electrolyte, almost all of the potential drop at the interface occurs within the Helmholtz region in the electrolyte phase. On the other hand, the interfacial potential drop across a semiconductor-electrolyte junction is partitioned into V_sc_ and V_H_, leading to a simple equivalent circuit model comprising two capacitors, C_sc_ and C_H_, in series (see Figure 3) [24,25].

### 5.1. Total Potential Difference Across the Interface

The relative positions of the solution energy levels with respect to the semiconductor band edge positions at the interface can be represented by the total potential difference [26]:

(45) $$V=V_{\mathrm{SC}}+V_{H}+V_{G}$$

where V is the electrode potential, as measured between an ohmic contact on the backside of the semiconductor electrode and a reference electrode. The potential difference, V_sc_, appears as a bending of the energy bands, as indicated in Figure 2, and the total capacitance, C, in series (see Figure 3) is given by:

(46) $$\frac{1}{C}=\frac{1}{C_{\mathrm{sc}}}+\frac{1}{C_{H}}+\frac{1}{C_{G}}$$

The problematic factors in placing the semiconductor and solution energy levels on a common basis involve V_H_ and V_G_. In other words, theoretical predictions of the magnitude of V_sc_ (and how it changes as the redox couple is varied) are hampered by a lack of knowledge of the magnitude of V_H_ and V_G_.

**Figure 4.**
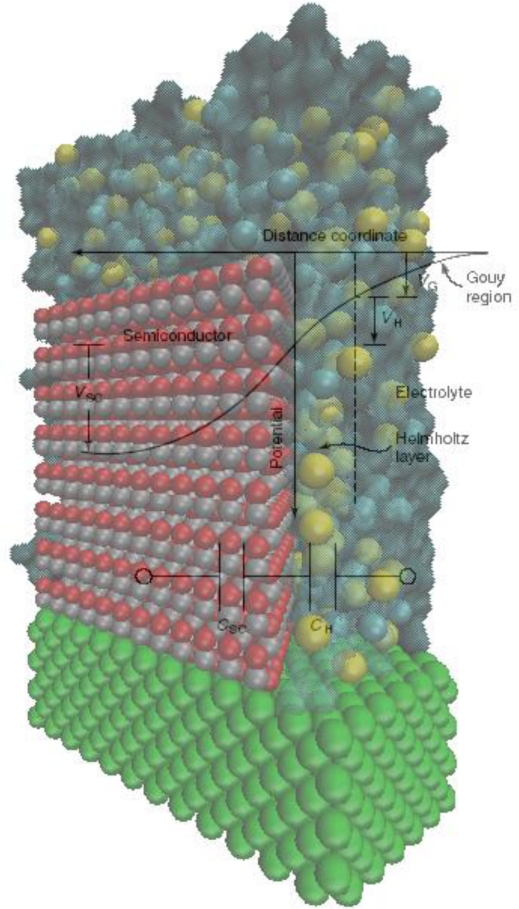
A simplified equivalent circuit for the semiconductor-electrolyte interface at equilibrium where the Gouy layer is neglected.

A degree of simplification is afforded by employing relatively concentrated electrolytes, such that V_G_ can be ignored [27] (see Figure 4):

(47) $$V=V_{\mathrm{sc}}+V_{H}$$

(48) $$\frac{1}{C}=\frac{1}{C_{\mathrm{sc}}}+\frac{1}{C_{H}}$$

As with metals, the Helmholtz layer is developed by adsorption of ions or molecules on the semiconductor surface, by oriented dipoles, or, especially in the case of oxides, by the formation of surface bonds between the solid surface and species in solution. Information on band edge placement can be sought through differential capacitance measurements on the semiconductor-redox electrolyte interface [28]. From the capacitor in series model, we can see that the semiconductor space charge layer is usually the determining factor in the total capacitance of the interface. The capacitance of the Helmholtz layer depends only very little on potential. On the other hand, the space charge semiconductor capacitance depends strongly on the potential.

The potential distribution in the space charge layer of a semiconductor can be found by solving the Poisson Equation for a given charge distribution [29,30]. For a semiconductor-electrolyte interface where the density of an electron donor, N_D_, is constant throughout the semiconductor, the potential, V(x), at a distance, x, from the surface is given as follows [29–31]:

(49) $$V\;(x)=\frac{{\mathrm{qN}}_{D}}{\varepsilon_{o}\;\varepsilon_{S}}\;\left(\mathrm{Wx}-\frac{1}{2}\;x^{2}\right)+V(0)\;\;\;\;\;\;\;\text{where}\;\;(0\leq x\leq W)$$

where q is the elementary charge, ε_o_ the permittivity of vacuum, ε_s_ the dielectric constant of the semiconductor, V(0) the potential at the surface (x=0) and the width of the space charge layer W. The values of V(0) and W are given by:

(50) $$V\;(0)=\frac{{\mathrm{qN}}_{D}W}{\varepsilon_{0}\;\varepsilon_{S}}\;\delta$$

(51) $$W={\left[\frac{2\varepsilon_{0}\;\varepsilon_{S}}{{\mathrm{qN}}_{D}}\left(V-V_{\mathrm{fb}}-\frac{\mathrm{kT}}{q}\right)\right]}^{\frac{1}{2}}$$

where δ is the thickness of the (outer) Helmholtz layer, V is the electrode potential, and V_fb_ is the flat band potential. Further reflection shows how the magnitude of W should depend on the semiconductor parameter N_D_, i.e., the thickness of the space charge layer decreases with increasing doping. For a typical carrier density of *n*_0_ = 10^17^ cm^−3^, and a band bending of V_sc_ = 0.5 V, one obtains W = 10^−5^ cm. Nominal dimensions of W are in the 10–1,000 nm range. This may be compared with the corresponding Helmholtz layer width, typically 0.4–0.6 nm.

### 5.2. The Differential Capacitance of the Space Charge Layer

The space charge, Q_s_, per unit area is given by [32]:

(52) $$Q_{s}={\mathrm{qN}}_{D}W={\left[2q\varepsilon_{0}\;\varepsilon_{s}\;N_{D}\left(V-V_{\mathrm{fb}}-\frac{\mathrm{kT}}{q}\right)\right]}^{\frac{1}{2}}$$

Thus, the differential capacitance of the space charge layer, C_sc_, per unit area is given as follows:

(53) $$C_{\mathrm{sc}}=\frac{\partial Q_{s}}{\partial U}={\left[\frac{2}{q\varepsilon_{0}\;\varepsilon_{S}\;N_{D}}\cdot \left(V-V_{\mathrm{fb}}-\frac{\mathrm{kT}}{q}\right)\right]}^{-\frac{1}{2}}$$

This Equation can be rewritten as follows:

(54) $$\frac{1}{C_{\mathrm{sc}}^{2}}=\frac{2}{q\varepsilon_{0}\;\varepsilon_{S}\;N_{D}}\left(V-V_{\mathrm{fb}}-\frac{\mathrm{kT}}{q}\right)$$

Equation (54) can be applied to non-degenerate semiconductor systems.

In the above discussion, the energy bands are pinned at the surface, and any variation of the electrode potential leads to a change in the band bending [33–35] (see Figure 2b). Investigations of many semiconductor electrodes have shown that the positions of the energy bands are independent of the doping. Therefore, the energy bands of n-type electrodes have the same position at the surface, as shown in Figure 1. As previously mentioned, in aqueous solutions, the potential across the Helmholtz double layer is entirely determined by the interaction of the semiconductor with the solvent. If the energy band edges are pinned, they do not shift upon changing the redox system. Only a change in band bending occurs to maintain equal Fermi levels on both sides of the interface. However, there are many cases where the energy bands are not pinned, but the Fermi level of the semiconductor is pinned [36,37].

**Figure 5.**
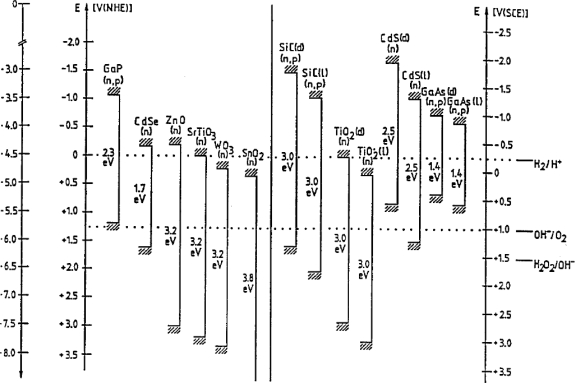
Position of energy bands of various semiconductors with respect to the electrochemical scale (adopted from Ref. [27]).

The energy positions at the surface for several semiconductors in contact with aqueous solutions are given in Figure 5. In many cases, the flat-band potential V_fb_, and consequently the position of the energy bands, varies with the pH of the solution because of protonation and deprotonation of surface hydroxyl groups. This effect is especially pronounced with oxide semiconductors, germanium and some III–V compounds.

### 5.3. Measurement of Differential Capacitance

The capacitance of the semiconductor-electrolyte interface can be measured by use of a semiconductor electrode, in which the front side of the semiconductor is in contact with the electrolyte and the rear side is electrically connected to a metallic wire via an ohmic contact. The differential capacitance is measured by superimposing an AC voltage, with a small amplitude of about 10 mV and a frequency of a few Hz to 1 MHz, on the electrode potential [38].

#### 5.3.1. Applied potential

**Figure 6.**
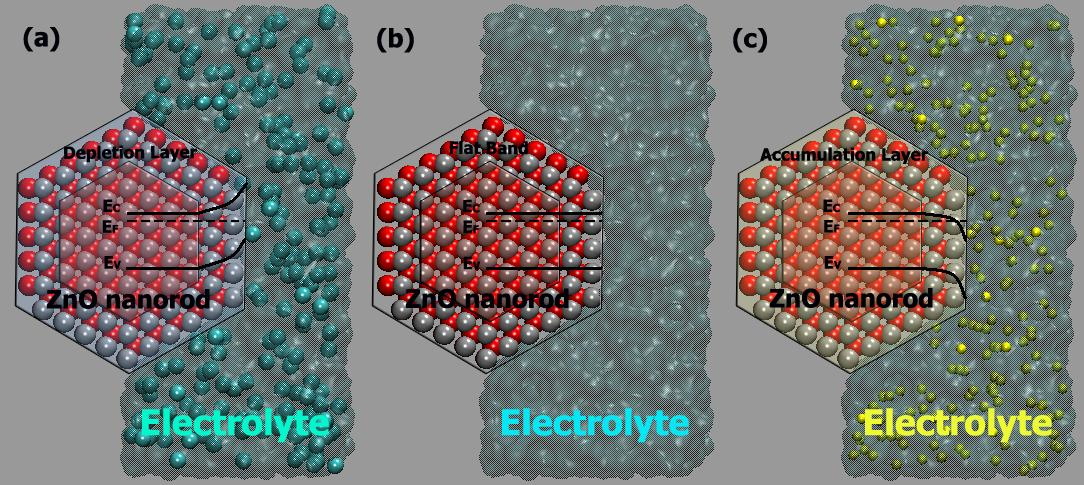
Effect of varying the applied potential E on the band edges in the interior of an n-type semiconductor where (a) E > E_fb_, (b) E = E_fb_, and (c) E < E_fb_.

For metallic electrodes, changing the applied potential shifts the Fermi level. The band edges in the interior of a semiconductor (i.e., away from the depletion region) vary with the applied potential in the same way as does the Fermi level in a metal. However, the energies of the band edges at the interface are not affected by changes in the applied potential. Therefore, the change in the energies of the band edges on going from the interior of the semiconductor to the interface, and hence the magnitude and direction of the band bending, varies with the applied potential. There are three different situations to be considered [39,40]:

1. At a certain potential, the Fermi energy lies at the same energy as the solution redox potential (see Figure 6b). There is no net transfer of charge, and there is no band bending. This potential is therefore referred to as the flatband potential (E_fb_).
2. For an n-type semiconductor, depletion regions arise at potentials positive of the flatband potential (see Figure 6a).
3. For n-type semiconductors at potentials negative of the flatband potential, there is now an excess of the majority charge carrier (electrons) in this space charge region, which is referred to as an accumulation region (see Figure 6c).

The charge transfer abilities of a semiconductor electrode depend on whether there is an accumulation layer or a depletion layer. If there is an accumulation layer, the behavior of a semiconductor is similar to that of a metallic electrode, since there is an excess of the majority charge carrier available for charge transfer. In contrast, if there is a depletion layer, then there are few charge carriers available for charge transfer, and electron transfer reactions occur slowly, if at all.

#### 5.3.2. Mott-schottky plots and flat band potentials

Using Equation (54), we can create a plot of 1/C_sc_^2^ measured against V. This plot is called the Mott-Schottky plot and gives a straight line with a slope of (2/qε_0_ε_s_N_D_). The extrapolation of the straight line to 1/C_sc_^2^ = 0 gives (V_fb_ + kT/q). Therefore, the plot can be used to determine the flat band potential, V_fb_. The donor density, N_D_, can also be determined from the slopes of these plots. It should be noted that the slope of the straight line depends not only on N_D_ and ε_s_ but also on the true surface area (or surface roughness) of the semiconductor electrode [41].

**Figure 7.**
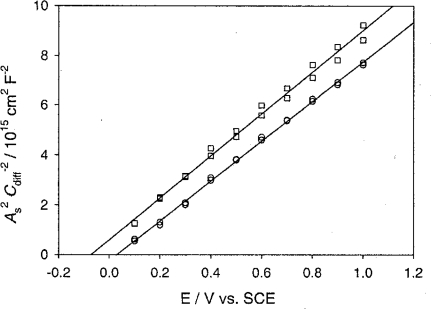
Mott-Schottky plots for n-type ZnO in contact with phthalate buffer solutions at pH 4 (circles) and pH 6 (squares) (adopted from Ref. [41]).

The flat-band potential, V_fb_ (the potential at which the bands of the semiconductor become flat, i.e., the potential at which charge in the semiconductor, Q_sc_, is zero), is one of the most important quantities for semiconductor electrodes because it determines the band edge positions at the semiconductor-electrolyte interface. These positions, in turn, determine the energies of conduction-band electrons and valence-band holes reacting with the electrolyte solution. It is known that, for most semiconductors such as n-ZnO in aqueous electrolytes, V_fb_ is solely determined by the solution pH and shifts in proportion to pH with a ratio of −0.059 V/pH [29,30] (see Figure 7). This phenomenon is explained by the adsorption equilibrium for H^+^ or OH^−^ at the semiconductor-electrolyte interface.

#### 5.3.3. Heavily doped n-type semiconductors (N_D_ > 10^20^ cm^−3^)

Taking into account the effect of the Helmholtz capacitor and C_sc_, the differential capacitance of the space charge layer per unit area is given as follows [42]:

(55) $$C^{-2}=C_{H}^{-2}\left[1+\left(\frac{4C_{H}^{2}}{\varepsilon_{0}\;\varepsilon_{s}\;{\mathrm{qN}}_{D}}\right)\;\left(V-V_{\mathrm{FB}}-\frac{\mathrm{kT}}{q}\right)\right]$$

As we can see from the above Equation, the relationship between C^−2^ and V is linear, and the slope is equal to (2/qε_0_ε_s_N_D_), the same value as in the simple Mott-Schottky relationship [Equation (54)].

However, the intersection with the V axis gives [42–44]:

(56) $$V_{o}=V_{\mathrm{FB}}+\frac{\mathrm{kT}}{q}-\frac{\varepsilon_{0}\;\varepsilon_{s}\;{\mathrm{qN}}_{D}}{4C_{H}^{2}}\;\;\text{at}\;\;C^{-2}=0$$

With respect to the value deduced from Equation (54), this value is shifted by the third term on the right side of the above Equation. The contrast with the corresponding metal-electrolyte interface is striking. The situation becomes similar to the metal-electrolyte interface only when the semiconductor is degenerately doped (N_D_ > 10^20^ cm^−3^, which leads to a rather large space charge layer charge, Q_sc_ and a thin depletion layer), or when its surface is in accumulation.

**Figure 8.**
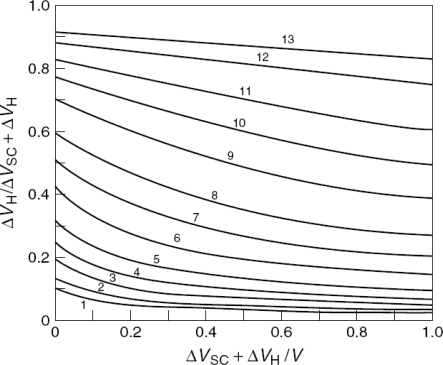
The ratio of the potential drop in the Helmholtz to the total potential change computed as a function of the total potential change. A static dielectric constant of 173 (typical of TiO_2_) and a Helmholtz capacitance of 10 μF/cm^2^ were assumed and the doping density was allowed to vary from 10^16^ cm^−3^ (curve 1) to 10^20^ cm^−3^ (curve 13) (adopted from Ref. [45]).

Finally, within the Mott-Schottky approximation [Equation (54)], large values of ε_s_ or N_D_ can lead to large values of the ratio V_H_/V_sc_. Figure 8 contains estimates of this ratio for several values of N_D_ for a semiconductor with a large ε_s_ value, mapped as a function of the total potential drop across the interface [45]. Clearly, V_H_ can become a sizable fraction of the total potential drop (approaching the situation for metals) under certain conditions. It has been shown [46] that, in this situation, the Mott-Schottky plots will still be linear, but the intercept on the potential axis is shifted from the V_fb_ value.

### 5.4. n-Type ZnO Nanowire-Electrolyte Interface

Single crystal ZnO nanowires have been used to determine the ZnO carrier density [47–52]. In these studies, Mott-Schottky analysis was used to determine both dopant density and flat-band potential at the ZnO-electrolyte contact. In flat electrodes, the capacitance per unit area of surface is:

(57) $$\frac{1}{C_{s}^{2}}=\frac{2}{q\varepsilon_{0}\;\varepsilon_{s}\;N_{D}}\;\left(V_{\mathrm{sc}}+V_{o}\right)$$

where V_sc_ is the potential difference across the semiconductor space charge region, and V_o_ takes into account the contributions of the Helmholtz layer and the flat band potential.

In the nanowire semiconductor structure, a circular depletion layer will grow from the surface towards the center of the wire with increasing bias. The geometry may introduce significant changes with respect to Equation (57). Ivan assumes that each nanowire is described as a cylinder of radius R with axial symmetry and a donor density N_D_. The Poisson Equation in cylindrical coordinates for one dimension is [49]:

(58) $$\frac{1}{r}\;\frac{\partial }{\partial r}\;\left(\frac{\partial V}{\partial r}\right)=-\frac{{\mathrm{qN}}_{D}}{\varepsilon_{o}\;\varepsilon_{s}}$$

**Figure 9.**
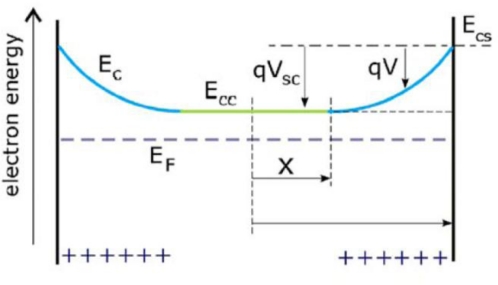
Schematic energy diagram in the radial direction of a nanowire indicating the depletion layer at the surface and the quasineutral region of radius x in the center where V is the potential, V_sc_ is the potential drop across the depletion layer, E_c_ is the lower edge of the conduction band, E_F_ is the Fermi level (adopted from Ref. [49]).

This Equation can be solved in the depletion approximation for the voltage, V. As shown in Figure (9), the central zone of the cylinder is a neutral region of radius x and electron density n = N_D_. The surface, defined as x ≤ r ≤ R, is a region of positive space charge, qN_D_. The reference, V = 0, is taken at the semiconductor surface.

The voltage in the quasineutral region, V_sc_, which coincides with the total voltage drop across the barrier, is:

(59) $$V_{\mathrm{sc}}=-\frac{{\mathrm{qN}}_{D}}{2\varepsilon_{o}\;\varepsilon_{s}}\;\left[\frac{1}{2}\left(R^{2}-x^{2}\right)+R^{2}\;\text{ln}\;\left(\frac{x}{R}\right)\right]$$

**Figure 10.**
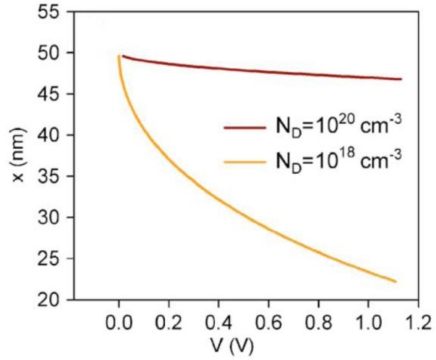
Simulation result of radius of the neutral region vs barrier voltage for different quantities for nanowire of radius R = 50 nm and length L = 1,000 nm, density of nanowires per flat unit area D_nw_ = 3 × 10^9^ cm^−2^, flat area S = 1 cm^2^, and ε_r_= 10, for two different donor densities N_D_ = 10^18^ and N_D_ = 10^20^ cm^−3^ (adopted from Ref. [49]).

Figure (10) shows the effect of N_D_ on x as function of V_sc_. A high donor density implies an ultra-thin space charge region in ZnO nanowires. For N_D_ = 10^20^ cm^−3^, this region is limited to less than 3 nm thickness. The reduction of N_D_ by two orders of magnitude causes a considerable portion of the nanowire to be depleted. It follows that nanowires with this radius are able to maintain band bending in the vicinity of their surface.

The positive charge in a cylinder of length L is:

(60) $$Q={\mathrm{qN}}_{D}\pi \left(R^{2}-x^{2}\right)L$$

Computing dV_sc_/dx and dQ/dx, we obtain the capacitance as a function of the radius of the neutral region. The capacitance per unit area of the cylinder surface is:

(61) $$C_{s}=\frac{1}{2\pi \mathrm{RL}}\frac{\mathrm{dQ}}{{\mathrm{dV}}_{\mathrm{sc}}}=\frac{2\varepsilon_{o}\;\varepsilon_{s}\;x^{2}}{R\left(R^{2}-x^{2}\right)}$$

**Figure 11.**
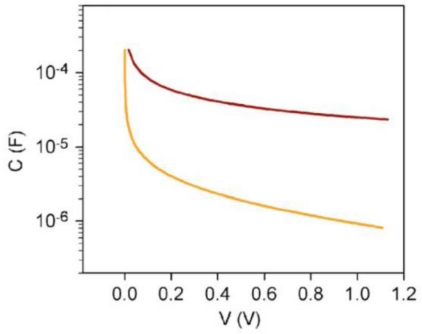
Simulation result of capacitance vs barrier voltage (adopted from Ref. [49]).

The behavior of the capacitance is illustrated in Figure (11).

**Figure 12.**
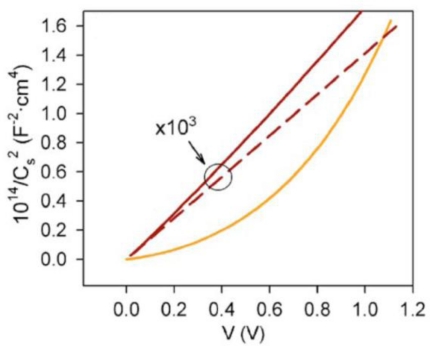
Simulation result of Mott-Schottky plot where the dashed line represents the expected Mott-Schottky using the conventional Equation for a flat interface with ND = 1,020 cm^−3^ (adopted from Ref. [49]).

**Figure 13.**
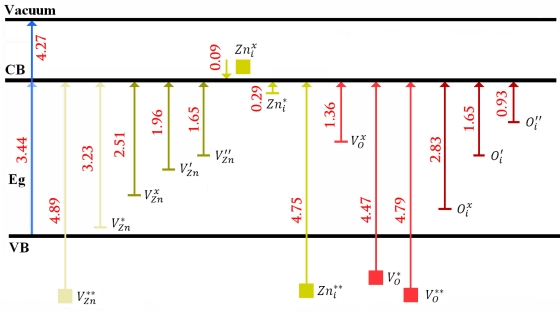
Energy levels of intrinsic defects in ZnO using Kröger Vink notation: i = interstitial site, Zn = zinc, O = oxygen, and V = vacancy. The terms indicate the atomic sites, and superscripted terms indicate charges, where a star indicates positive charge, a prime indicates negative charge, and a cross indicates zero charge, with the charges in proportion to the numbers of symbols (values of energy in eV).

The Mott-Schottky plot for an array of nanowires does not exhibit the linear behavior of Equation (57) observed in flat samples. The deviation from linear behavior decreases as N_D_ increases. It can be shown that, in the low voltage limit, i.e., x→R and V_sc_→0, the Mott-Schottky plot of a cylindrical sample tends to Equation (57) (see Figure 12). The increase of N_D_ of the nanowire could be due to an increase in the number of defects that produce electrically activated donor levels, such as interstitial Zn atoms (Zn_i_) and/or O vacancies (V_O_) [53] (see Figure 13).

## 6. n-Type Semiconductor-Electrolyte Interfaces (with Surface States)

**Figure 14.**
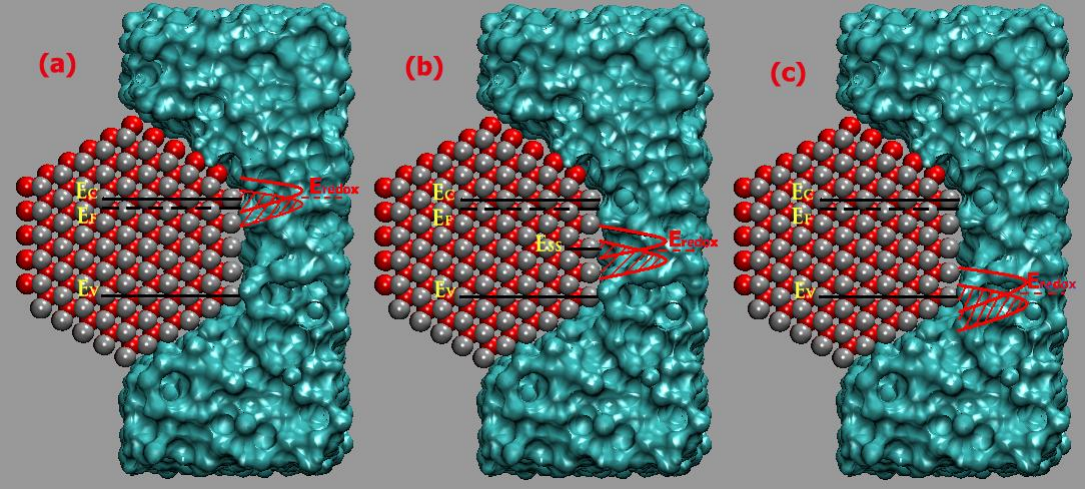
Three situations for a n-type semiconductor-electrolyte interface at equilibrium showing overlap of the redox energy levels with the semiconductor E_C_ (a), with surface states (b), and with the semiconductor E_V_ (c), where the discrete energy level is assumed for the surface states as a first approximation.

Surface states arise because of the abrupt termination of the crystal lattice at the surface, resulting in a bonding arrangement that is different from that in the bulk (dangling bonds). Consider our prototypical semiconductor, ZnO. The tetrahedral bonding characteristic of the bulk gives way to coordinative unsaturation of the bonds for the Zn and O surface atoms. This unsaturation is relieved either by surface reconstruction or bonds with extraneous (e.g. solvent) species [54]. The surface bonding results in a localized electronic structure for the surface that is different from that of the bulk. The energies of these localized surface orbitals nominally lie in the forbidden band gap region. The corresponding states are thus able (depending on their energy location) to exchange charge with the conduction band of the semiconductor and/or the redox electrolyte [55] (see Figure 14).

Changes in the solution redox potential have been observed to cause no change in the magnitude of V_sc_. This situation is termed Fermi level pinning. In other words, the band edge positions are unpinned in these cases, so that the movement of E_redox_ is accommodated by V_H_, rather than V_sc_. It appears [56] that surface state densities as low as 10^13^ cm^−2^ (≈1% of a monolayer) suffice to induce complete Fermi level pinning in certain cases. Of course, intermediate situations are also possible. The ideal case manifests a slope of 1 in a plot of V_sc_ (or an equivalent parameter) versus E_redox_. On the other hand, complete pinning results in a slope of zero. Intermediate cases of Fermi level pinning exhibit slopes between 0 and 1 [57].

There are two cases for the treatment of a semiconductor/liquid interface in the presence of surface states [58]. First, if there are a large number of states between the valence and conduction band and these states extend throughout the semiconductor, resulting in a continuum of states, the semiconductor is metal-like in its electrode behavior. Such materials are associated with so-called degenerate doping, which provides so many charge carriers that a space-charge region inside the semiconductor is not possible. The behavior of such materials is metal-like. When electronic equilibrium occurs between the electrode and the solution, the potential drop occurs exclusively across the Helmholtz layer at the interface. Variation of the potential between the bulk semiconductor and the solution results in changes in the potential drop across the Helmholtz layer and not within the low-resistance semiconductor. Second, the semiconductors may have a significant density of surface states between E_VB_ and E_CB_ that can exhibit Fermi level pinning when contacting a liquid electrolyte solution. In such a system, (1) many redox couples having different electrochemical potentials give the same output voltage, (2) two couples whose formal potentials are more widely spaced than the separation of E_CB_ and E_VB_ for a given semiconductor give comparable output voltage, and (3) surface modification aimed at changing the number and location of surface states may be an important way to improve the output characteristics of these electrochemical devices, where Fermi level pinning dominates the properties of the electrode.

### 6.1. Fermi Level Pinning

Fermi level pinning in semiconductor/metal Schottky barriers refers to the phenomenon in which the surface states of a semiconductor give rise to a fixed barrier height, independent of the meta1 used [59–62]. For some semiconductors, it has been determined that the Schottky barrier height is independent of the metal, even for metals having very large differences in work function. However, when the barrier height is “pinned” to a constant value, it is believed that surface states between E_VB_ and E_CB_ must be taken into account. The phenomenon of a metal-insensitive barrier height is referred to as Fermi level pinning and is believed to result from a significant density of surface states at a defined potential. The Fermi level becomes pinned to these states, independent of the overlying metal. Fermi level pinning contributes to the Schottky barrier since the output voltage is limited to a value determined by the surface states of the semiconductor. If Fermi level pinning occurs for certain semiconductor/liquid junctions, we can treat these junctions as Schottky barriers, and attribute the effect to a significant density of surface states that are localized to a certain potential. Thus, the surface states play a role analogous to that in a semiconductor-metal junction. The density and energy distribution of surface states determines their energy level or work function, just as they would for a metal. The result is a semiconductor whose degree of band bending (barrier height) is determined by the layer of surface states. When this is the case, the shift of the flat band potential is due to an unpinning of energy bands. Minority carriers accumulate at the surface, which leads to a change in the potential across the Helmholtz double layer, i.e., ΔV_fb_ = V_H_. Band edge unpinning can also occur if carrier inversion develops at the semiconductor electrode surface [63].

**Figure 15.**
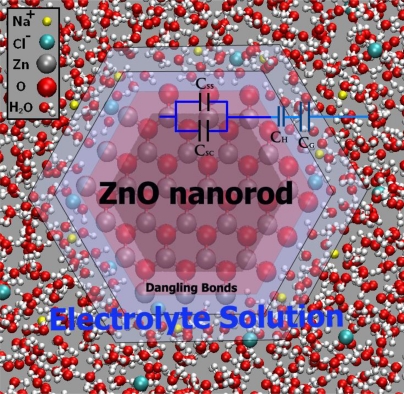
Approximate equivalent circuits for the n-type ZnO nanorod-liquid interface in presence of surface states. C_SS_ is the surface states capacitance, C_SC_ is the semiconductor depletion layer capacitance, C_H_ is Helmholtz layer capacitance, and C_G_ is Gouy capacitance.

In this situation, changes in the Fermi level of the system, due to either changes in the redox system or changes in the applied potential, will shift the energy bands. The total capacitance C (see Figure 15) is given by:

(62) $$\frac{1}{C}=\frac{1}{C_{\mathrm{sc}}+C_{\mathrm{ss}}}+\frac{1}{C_{H}}+\frac{1}{C_{G}}$$

where the total potential drop across the interface, V, caused by excess charge is the sum of the potential drops across the capacitors in series:

$$V=V_{\mathrm{sc}}+V_{H}+V_{G}$$

The potential drop across C_sc_ is the same as the potential drop across C_ss_ (as these capacitors are in parallel).

Qualitatively, Fermi level pinning will be important when the charge in the surface states (Q_ss_) becomes appreciably larger than that in the space charge region (Q_sc_). Under these conditions, changes in potential between the bulk semiconductor and the bulk solution will mainly affect the potential drop across the Helmholtz layer, V_H_, rather than the drop within the semiconductor (V_sc_). In this case, even in the absence of an electrolyte solution, band bending within the semiconductor can occur (Q_El_ = 0, Q_sc_ = −Q_ss_) [64,65]. For a semiconductor with ionized surface states, the condition of charge neutrality will be:

(63) $$Q_{\mathrm{sc}}=Q_{\mathrm{El}}+Q_{\mathrm{ss}}$$

And, according to the Fermi-Dirac distribution, for singly ionizable surface states, the surface excess charge concentration, Q_ss_, is given by [58,22]:

(64) $$Q_{\mathrm{ss}}=\frac{{\mathrm{qN}}_{\mathrm{ss}}}{\left[1+g_{\mathrm{ss}}^{-1}\;e^{\frac{-\left[\left(E_{\mathrm{ss}}-E_{F}\right)-{\mathrm{qV}}_{\mathrm{sc}}\right]}{\mathrm{kT}}}\right]}$$

where (*E_ss_* – *E_F_*) is the energy interval between the surface states and the Fermi level when V_sc_ = 0. If the energy bands of the semiconductor are not bent, g_ss_ is the degeneracy of the energy level, and N_ss_ is the surface concentration of donor states. Given any assumed value of V, if the surface state energy and concentration are known, the individual values of V_sc_, V_H_, and V_G_ can be calculated. Thus both Q_sc_ and Q_ss_ are functions of V_sc_. For any given value of V_sc_, Q_H_ can be obtained using Equation (63).

In the presence of surface states, the relation between Q_sc_ and Vsc depends upon the distribution of surface state energies (whether the surface states are uniformly distributed in energy [59] or are localized at a single energy level [66]). For example, if a uniform distribution of acceptor surface states centered on an energy E_o_ is assumed, there is no net surface charge when the states are filled to an energy E_o_. If Q_El_ is known, V_G_ can be obtained. Similarly, knowledge of Q_El_ gives the value of V_H_. The essential point which emerges from numerical solutions for semiconductor-electrolyte electrodes with a high concentration of ionized surface states (e.g. 10^15^ cm^−2^, 1/10 ionized) is that V_H_ may be large (e.g. 1 V). Therefore, for the case of a high concentration of ionized surface states, a semiconductor-electrolyte electrode behaves like a metal-electrolyte electrode. If a potential difference is applied across such a semiconductor-electrolyte electrode and the sum of all changes in potential drops is equal to V, then V_sc_ will remain almost the same, while V_H_ and V_G_ will change. Such behavior is apparent from the fact that a slight shift in V_sc_ will cause the ionization or deionization of many surface states. This ionization or deionization will, in turn, change Q_ss_ and hence V_H_. In this case, the semiconductor-electrolyte electrode behaves like a metal-electrolyte electrode. Note that, within the scope of this treatment, no distinction can be drawn between surface states inherent to the semiconductor surface (“inside” the semiconductor surface) attributable to dangling bonds, surface imperfections, etc. (Tamm [67] or Shockley [68] states), and those formed at the semiconductor surface by adsorption of electron acceptor or donor molecules or by surface modification by intentional attachment of electroactive functionalities. However, ionizable surface groups (groups in which charge is produced by a chemical reaction such as deprotonation) or adsorbed ions can be treated separately, as pointed out by Gerischer [69].

### 6.2. Electrochemical Potential of n-Type ZnO Nanorod Electrodes (Potentiometric Measurements)

Several types of electrodes are used in electrochemical measurements. For this work, we used single-crystal ZnO nanorods as an electrochemical potential pH electrode. The Helmholtz layer is developed by adsorption of ions or molecules on the ZnO nanorod surfaces, by oriented dipoles, or by the formation of surface bonds between the solid surface and species in solution. Water dissociates into hydronium *H*_3_*O*^+^ and hydroxyl *OH*^−^ ions, but for simplicity we refer to the hydronium ion as a hydrogen ion *H*^+^ in chemical reaction Equations. The metal atoms in an amphoteric oxide must be fairly electropositive to give the oxygen sufficient negative charge to strip a proton from a neighboring *H*_3_*O*^+^. However, the metal ion must also be electronegative enough to serve as an electron acceptor from a neighboring *OH*^−^. For amphoteric ZnO surfaces, we can write local equilibrium reactions and equilibrium constants:

(65) $$\mathrm{Zn}{\left(\mathrm{OH}\right)}_{2(S)}+H_{\left(\mathrm{aq}\right)}^{+}={\mathrm{ZnOH}}_{\left(\mathrm{aq}\right)}^{+}+H_{2}O\;\;\;\;\text{for acid}$$

(66) $${\mathrm{ZnO}}_{2}H_{\left(\mathrm{aq}\right)}^{+}+H_{\left(\mathrm{aq}\right)}^{+}=\mathrm{Zn}{\left(\mathrm{OH}\right)}_{2(S)}\;\;\;\;\;\;\text{for base}$$

where K_1_ and K_2_ are the dissociation constants, which can be estimated from known thermodynamic data for equilibria of the reactions:

(67) $$K_{1}=\frac{\left\lfloor {\mathrm{ZnOH}}^{+}\right\rfloor }{\left[\mathrm{Zn}{\left(\mathrm{OH}\right)}_{2}\right]\;\left[H^{+}\right]}$$

(68) $$K_{2}=\frac{\left[\mathrm{Zn}{\left(\mathrm{OH}\right)}_{2}\right]}{\left[{\mathrm{ZnO}}_{2}H^{+}\right]\;\left[H^{+}\right]}$$

If these ZnO nanorods are degenerately doped (N_D_ > 10^20^ cm^−3^), they possess a rather large space charge layer charge, Q_sc_ and a thin depletion layer. In this instance, the situation becomes similar to a metal-electrolyte interface. The increase in N_D_ in the nanorods could be due to an increase in the number of defects that produce electrically activated donor levels, such as interstitial Zn atoms (Zn_i_) and/or O vacancies (V_O_). When electronic equilibrium occurs between the electrode and the solution, the potential drop occurs exclusively across the Helmholtz layer at the interface. The electrochemical potential difference between ZnO nanorods and the solution results from changes in the potential drop across the Helmholtz layer and not within the low-resistance ZnO nanorod. We interpret this result as Fermi level pinning by surface states. At pH < 9, on the first contact of ZnO nanorods with the aqueous solution, adsorption of H^+^ on ≡ZnOH groups near the surface takes place. At pH > 9, on the first contact of ZnO nanorods with the aqueous solution, ≡ZnOH groups combine with *OH*^−^ ions, forming ≡*ZnO*^−^. In terms of solid state chemistry, ≡ZnOH^+^ and ≡*ZnO*^−^ groups form surface states.

During electrochemical potential pH measurements, the ZnO nanorods are positively charged, which provides a suitable environment for adsorption of low isoelectric point groups. Because of this suitability, we will use the chemical reaction in Equation (65) to find the electrochemical potential Equation for our ZnO nanorod sensor. The electrical potential difference, ΔΦ, between the electrolyte solution and the ZnO nanorods can be expressed as:

(69) $$\Delta \Phi =\Phi_{\mathrm{El}}-\Phi_{\mathrm{MO}}=\frac{1}{\mathrm{nF}}\;\left(\mu_{{\mathrm{ZnOH}}^{+}}+\mu_{H_{2}O}-\mu_{\mathrm{Zn}{\left(\mathrm{OH}\right)}_{2}}-\mu_{H^{+}}\right)$$

where Φ*_El_* is the electrical potential of the electrolyte solution, Φ*_MO_* is the electrical potential of the inert ZnO nanorod electrode, *n* is the number of electrons in the redox reaction, and *μ_i_* is the chemical potential of species *i* .

Equation (69) may thus be rewritten in the form of the Nernst Equation for the simple redox electrode:

(70) $$\Delta \Phi =E_{\mathrm{Zn}{\left(\mathrm{OH}\right)}_{2}\;|\;{\mathrm{ZnOH}}^{+}}=\frac{1}{\mathrm{nF}}\;\left(\mu_{{\mathrm{ZnOH}}^{+}}^{o}-\mu_{\mathrm{Zn}{\left(\mathrm{OH}\right)}_{2}}^{o}-\mu_{H^{+}}^{o}\right)+\frac{\mathrm{RT}}{\mathrm{nF}}\;\text{ln}\;\left(\frac{a_{{\mathrm{ZnOH}}^{+}}}{a_{\mathrm{Zn}{\left(\mathrm{OH}\right)}_{2}}\cdot a_{H^{+}}}\right)$$

where $\mu_{i}^{o}$ is the standard chemical potential of species *i* at unit activity *a_i_* = 1, and *a_i_* is the activity of the particular ions. For the redox system employed here, we obtain:

(71) $$E_{\mathrm{Zn}{\left(\mathrm{OH}\right)}_{2}\;|\;{\mathrm{ZnOH}}^{+}}^{o}=\frac{1}{\mathrm{nF}}\;\left(\mu_{{\mathrm{ZnOH}}^{+}}^{o}-\mu_{\mathrm{Zn}{\left(\mathrm{OH}\right)}_{2}}^{o}-\mu_{H^{+}}^{o}\right)$$

where *E^o^* is the standard electrode potential of the ZnO nanorod redox electrode.

(72) $$E_{\mathrm{Zn}{\left(\mathrm{OH}\right)}_{2}\;|\;{\mathrm{ZnOH}}^{+}}=E_{\mathrm{Zn}{\left(\mathrm{OH}\right)}_{2}|\;{\mathrm{ZnOH}}^{+}}^{o}+\frac{\mathrm{RT}}{\mathrm{nF}}\;\text{ln}\;\left(\frac{a_{{\mathrm{ZnOH}}^{+}}}{a_{\mathrm{Zn}{\left(\mathrm{OH}\right)}_{2}}\cdot a_{H^{+}}}\right)$$

At ideal dilution, if the activity, *a_i_*, is identical to the concentration, *c_i_*, then *a_i_* *≈* *c_i_* *=* [*i*]. The electrochemical potential of the ZnO nanorod electrode will be:

(73) $$E_{\mathrm{Zn}{\left(\mathrm{OH}\right)}_{2}\;|\;{\mathrm{ZnOH}}^{+}}=E_{\mathrm{Zn}{\left(\mathrm{OH}\right)}_{2}\;|\;{\mathrm{ZnOH}}^{+}}^{o}+\frac{\mathrm{RT}}{\mathrm{nF}}\;\text{ln}\;\left(\frac{\left\lfloor {\mathrm{ZnOH}}^{+}\right\rfloor }{\left[\mathrm{Zn}{\left(\mathrm{OH}\right)}_{2}\right]\cdot \left[H^{+}\right]}\right)$$

The value of *RT/F* is approximately equal to 25.684 mV. For n = 1, the expression above (73) can be simplified to give:

(74) $$E_{\mathrm{Zn}{\left(\mathrm{OH}\right)}_{2}\;|\;{\mathrm{ZnOH}}^{+}}=E_{\mathrm{Zn}{\left(\mathrm{OH}\right)}_{2}\;|\;{\mathrm{ZnOH}}^{+}}^{o}+(0.05915)\;\text{log}\left(\frac{\left\lfloor {\mathrm{ZnOH}}^{+}\right\rfloor }{\left[\mathrm{Zn}{\left(\mathrm{OH}\right)}_{2}\right]}\right)+\left(0.05915\right)\;\;\text{lg}\left(\frac{1}{\left[H^{+}\right]}\right)$$

The potential of the ZnO nanorod pH electrode is:

(75) $$E_{\mathrm{Zn}{\left(\mathrm{OH}\right)}_{2}\;|\;{\mathrm{ZnOH}}^{+}}=E_{\mathrm{Zn}{\left(\mathrm{OH}\right)}_{2}\;|\;{\mathrm{ZnOH}}^{+}}^{o}+(0.05915)\;\text{log}\left(\frac{\left\lfloor {\mathrm{ZnOH}}^{+}\right\rfloor }{\left[\mathrm{Zn}{\left(\mathrm{OH}\right)}_{2}\right]}\right)+\left(0.05915\right)\;\text{pH}$$

with −log(*a*_*H*^+^_) = *pH*. The last term on the right hand side of Equations (74) and (75) suggests that it is possible to use these electrodes as Nernstian pH sensors.

**Figure 16.**
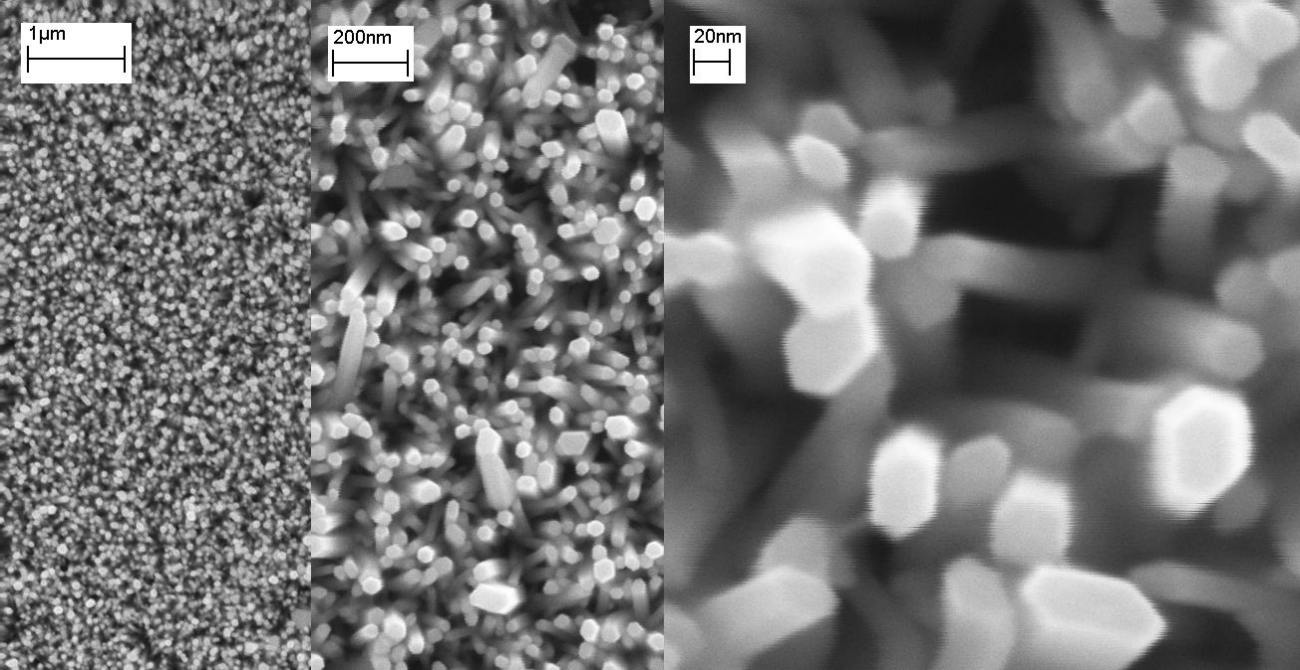
SEM images of the ZnO nanorods grown on Ag-coated n-Si substrate using low temperature growth (different magnification of the same sample).

The electrochemical device used here consists of a *Ag_(s)_*∣*AgCl_(s)_*∣*Cl^−^* electrode as a reference electrode. This reference supplied a constant potential, *E_Ag/AgCl/Cl_^−^*, against which we measured the potential of the ZnO nanorod redox electrode. We fabricated the ZnO nanorod sensor by growing hexagonal, single-crystal ZnO nanorods on silver-coated substrates using a low-temperature growth method described previously [70–72] (see Figure 16).

The resultant nanostructure is rod–shaped with a hexagonal cross section and is primarily aligned perpendicular to the substrate, a typical morphology of the wurtzite ZnO structure. The nanorods are uniform in size, with a diameter of 20–60 nm and a length of 500 nm [73]. The electrochemical device in this study can be represented as:

(76) $$\mathrm{Ag}|{\mathrm{AgCl}}_{\left(s\right)}|{\mathrm{Cl}}^{-}\vdots H_{2}O|{\mathrm{ZnOH}}^{+}|\mathrm{Zn}{\left(\mathrm{OH}\right)}_{2}$$

The device e.m.f. (*E*) is the potential difference between the supplied potential of the *ZnO* redox working electrode, *E_ZnO/ZnOH_^+^*, and the supplied potential of the standard reference electrode, *E_Ag/AgCl/Cl_^−^*:

(77) $$E=E_{\mathrm{Zn}{\left(\mathrm{OH}\right)}_{2}\;|\;{\mathrm{ZnOH}}^{+}}-E_{\mathrm{Ag}|\mathrm{AgCl}|{\mathrm{Cl}}^{-}}=E_{\mathrm{Zn}{\left(\mathrm{OH}\right)}_{2}\;|\;{\mathrm{ZnOH}}^{+}}^{o}+(0.05915)\;\text{log}\;\left(\frac{\left\lfloor {\mathrm{ZnOH}}^{+}\right\rfloor }{\left[\mathrm{Zn}{\left(\mathrm{OH}\right)}_{2}\right]}\right)+\left(0.05915\right)\cdot \mathrm{pH}-E_{\mathrm{Ag}|\mathrm{AgCl}|{\mathrm{Cl}}^{-}}$$

**Figure 17.**
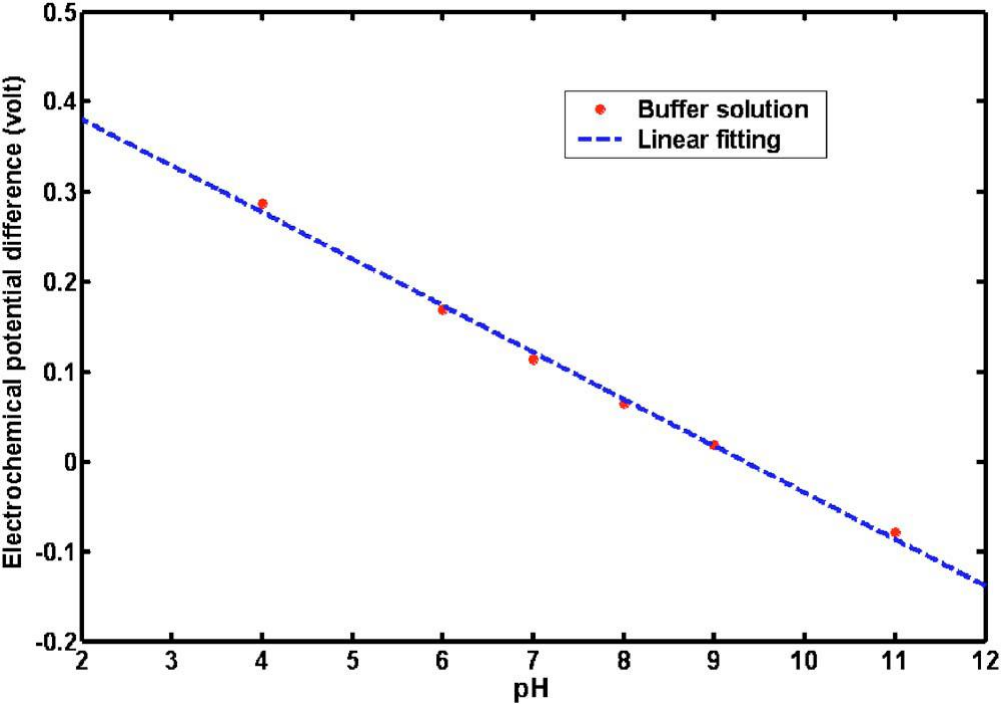
Calibration curve showing the electrochemical potential difference for ZnO nanorods as a working electrode with a Ag/AgCl reference microelectrode vs pH changes for buffer solutions (adopted from Ref. [73]).

We used a two-electrode configuration and milliliter sample volumes in these electrochemical studies. The ZnO nanorods functioned as the working electrode, and we used a standard Ag/AgCl reference electrode. All electrochemical experiments were conducted using a Metrohm model 827 pH meter at room temperature (22 ± 2 °C). We measured the electrochemical potential response of the ZnO nanorods (as a working electrode versus the Ag/AgCl reference electrode) to changes between standard buffers at room temperature. The results of this experiment show that this pH dependence is linear and has a sensitivity of 51.881 mV/pH at 22 °C (see Figure 17 [73]).

## 7. n-Type ZnO Sensing Mechanism

### 7.1. Surface Composition and Variation in PZC

The observed variations in point of zero charge (pzc) between the different zinc oxide precipitates may be related to changes in surface composition. It is known that anionic impurities generally lower the pzc of many oxides [74–76]. Parks explained this observation qualitatively by postulating that the surface group M–anion–H is a stronger acid than the surface group M–O–H, where M designates the metallic cation [74]. It would be more satisfactory to compare, for example, the surface states M–C1^…^H and M–OH^…^H and to ascribe the stronger acid-like nature of the former to an increased polarization of the larger anion and the inability of the chloride atom to hydrogen bond water molecules. ZnO that was prepared in a highly alkaline solution with excess base showed a pzc greater than pH 9.5, whereas samples formed at a less basic pH with a large excess of NO_3_^−^ had a pzc close to pH 8. The pzc values of other precipitates that should have impurity contents intermediate to these extremes were observed to vary between pH 8.5 and pH 9.5.

Anionic impurities, if present, should be leached out of the surface during a refluxing operation, and the pzc of the leached precipitate should fall at a higher pH than that of the unleached precipitate. The existence of a solid solution of Zn(OH)_2_ and Zn(OH)_1.6_X_0.4_ on the surface of zinc oxide has been postulated in order to explain the observed behavior in pzc. This composition model of the surface layer makes it possible to interpret the ion exchange process involving the anionic species *OH*^−^ and *X*^−^. It also helps to explain, qualitatively at least, the observed dependence of the pzc on the method of preparation of various zinc oxides [75].

### 7.2. Interface Mechanism

**Figure 18.**
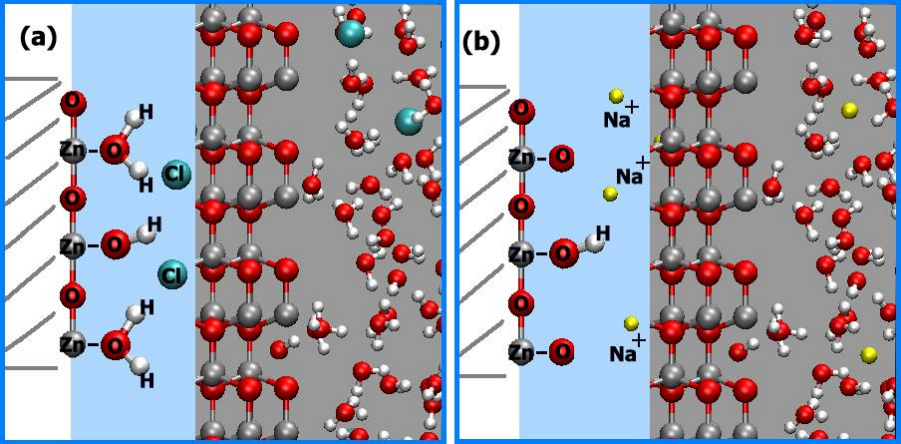
Schematic representation of the distribution of charges due to the slow reaction: (a) at pH < 8.9, (b) at pH > 8.9.

The diffusing species in the solid might be H^+^ at pH < 9 and OH^−^ at pH > 9. H^+^ ions should have a much higher diffusivity in ZnO than OH^−^. This characteristic might not be observed, however, if OH groups exist in the bulk of the initial ZnO at point defects. At pH > 9, the protons of such groups could diffuse out of the ZnO. The objection that charge would accumulate in the solid by such a process might be answered by assuming simultaneous transfer of counterions (Cl^−^ at pH < 9, Na^+^ at pH > 9) to the region behind the electrokinetic slipping plane. However, such diffusion would be influenced by the electric potential generated. If the counterions remain outside the solid (or penetrate the solid only at dislocations), whereas H^+^ diffuses into and out of undisturbed regions of ZnO, we could find the surface potential at the boundaries of these undisturbed regions by solving Poisson's relation (for a flat surface see section 5.1, for the cylinder surface see section 5.4). The proton concentration in ZnO decreases to 1/e of its surface concentration well within one unit cell, which excludes a diffusion-controlled process. Simultaneous diffusion of equimolar quantities of H^+^ and Cl^−^ into the ZnO at pH < 9, or exchange of Na^+^ against H^+^ at pH > 9, would remove this discrepancy. In order to be sterically possible, such a process should be restricted to dislocations. At the first contact of ZnO with an aqueous solution, adsorption of H^+^ and OH^−^ occurs (indicated as “primary adsorption”). This process is saturated at a relatively low degree of surface coverage. In addition, a reaction of surface ≡*Zn*–*OH* groups takes place with H^+^ ions at pH < 9 and with OH^−^ ions at pH > 9. The rate of this reaction is proportional to the concentration of free electrons or holes (vacant states in the valence band) at the surface. The charge, transferred by this process to the solid, does not remain at the surface itself but is distributed by electron transport over the near-surface region of the solid. This process forms a depletion layer or counteracts an accumulation layer at pH < 9 and forms or enhances an accumulation layer at pH > 9 (see Figure 18) [77].

**Figure 19.**
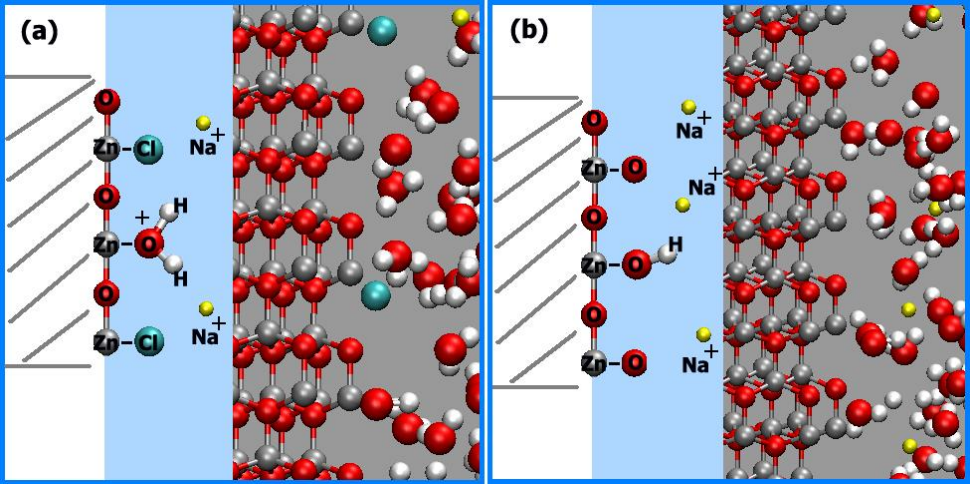
Schematic representation of the charges transferred to the ZnO by primary adsorption and chemisorption: (a) at pH < 8.9, (b) at pH > 8.9.

The surface charges are generated by primary adsorption and by chemisorption of *Cl*^−^. This process does not involve transport of Na^+^ or *Cl*^−^ ions into the solid (except in dislocations and then not as the rate-determining step). Proportionality of the reaction rate with the free electron concentration near the surface requires proportionality with band bending. The surface charge transferred at any pH by primary adsorption and chemisorption to the ZnO can be calculated from the reduced surface potential in the solid due to primary adsorption and chemisorption of *Cl*^−^ ions. The surface charges are negative at all pH values, since all surface potential values are positive. This relationship can be understood by the following mechanisms [77]:

- At pH < 9: On the first contact of ZnO with the aqueous solution, adsorption of H^+^ on ≡ZnOH groups and chemisorption of *Cl*^−^ on ≡Zn ions near the surface takes place. Of the charges transferred to the ZnO by these processes, only the negative charges imparted by *Cl*^−^ chemisorption are mobile (ZnO is an electronic semiconductor). These charges thus distribute themselves over the surface region (Figure 19a).
- At pH > 9: On the first contact of ZnO with the aqueous solution, ≡ZnOH groups combine with *OH*^−^ ions, forming ≡*ZnO*^−^. Again, the negative charge imparted by this process to the ZnO forms a space charge region (Figure 19b).

**Figure 20.**
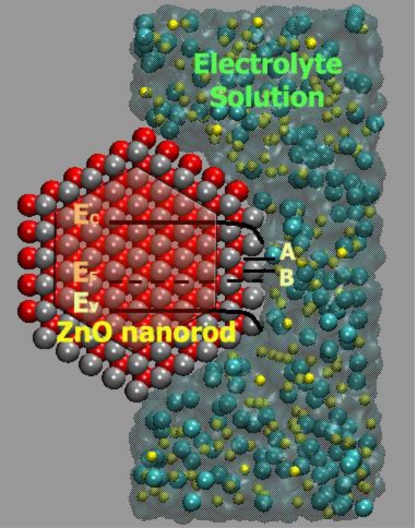
Schematic representation to the situation of the energy levels ZnO and surface states levels for ≡ZnC1^−^ and ≡ZnO^−^ groups which are labels as A and B, respectively.

In terms of solid state chemistry, ≡ZnCl^−^ and ≡ZnO^−^ groups form surface states whose energy levels should be situated as depicted in Figure 20 at A and B, respectively. Thus, in all cases, an accumulation layer is formed at the surface of the ZnO. In spite of the fact that free electrons at the surface are consumed by the reaction with H^+^ at pH < 9, there remains an accumulation layer throughout the conditions investigated.

#### 7.2.1. Adsorption of Water on the (0001)–*Zn* and (0001̄) – *O* Surfaces

ZnO crystallizes in the wurtzite structure, which does not have a center of inversion. Consequently, when the crystal is cleaved normal to the c axis in a manner that breaks the fewest interatomic bonds, two different polar surfaces are formed on opposite sides of the crystal. Each of these surfaces has only one type of ion. Thus, opposing surfaces bear an opposite charge on their outermost planes. The ideal polar surfaces are called (0001)*–Zn* when the Zn cation is present at the surface and (0001̄) – *O* when the *O* anion comprises the outermost layer. These polar ZnO surfaces are only stable if the (0001̄) – *O* face is less negative than, and the (0001)*–Zn* face is less positive than the formal bulk oxidation state by a factor of R_1_ / (R_1_ + R_2_) = 1/4, where R_1_ = 0.61 A and R_2_ = 1.99 A. This charge redistribution may be explained by a decrease in the ionic charge of the surface ions, from ±2 to ±3/2, which may be considered an electron transfer from the O-face to the Zn-face. As a result, partially occupied surface bands will appear with a ¾ filled O-2p band at (0001̄) – *O* and a ¼ filled Zn-4s band at the (0001)*–Zn* surface. This phenomenon is referred to as intrinsic state compensation [78] or as metallization of the polar surfaces [79].

**Figure 21.**
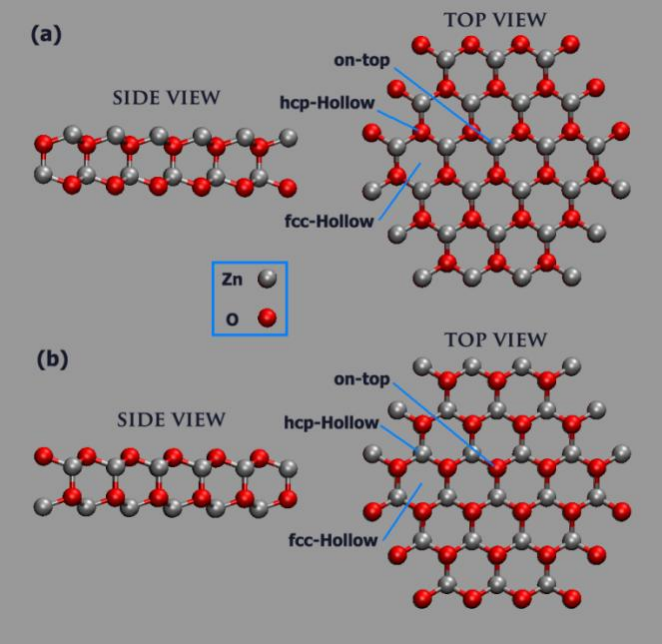
Schematic diagram of the high-symmetry adsorption sites on the two polar surfaces of ZnO, (a) for zinc surface termination top and side view, (b) for oxygen surface termination top and side view.

If the metallic state is present, it will depend on the dispersion of the partially occupied bands. In addition, the surface may reconstruct and undergo a distortion in which, for instance, at the (0001̄) – *O* surface, four surface atoms combine in such a way that an unoccupied 2p-band splits from the other eleven occupied 2p-bands and the surface become insulating again. On the other hand, the charge reduction of the surface layers may be take place by removing ¼ of the surface ions, creating vacancies. These vacancies may be ordered and may form a reconstruction, or they may be randomly distributed. When these ZnO polar surfaces come into contact with an electrolyte, the water molecules may dissociate and protons (H^+^) and hydroxyl groups (*OH*^−^) could adsorb, respectively, on every second O and Zn surface ion. The adsorption of these charged species would reduce the formal oxidation state of the surface ions. The adsorption sites of the dissociating water (*OH*^−^ and H^+^) on the polar surface are on-top positions, hcp-hollow site positions above atoms in the second surface layer, and a fcc-hollow site with no atoms beneath (see Figure 21) [80,81].

#### 7.2.2. Adsorption of Water on the (101̄0)-ZnO Surfaces

**Figure 22.**
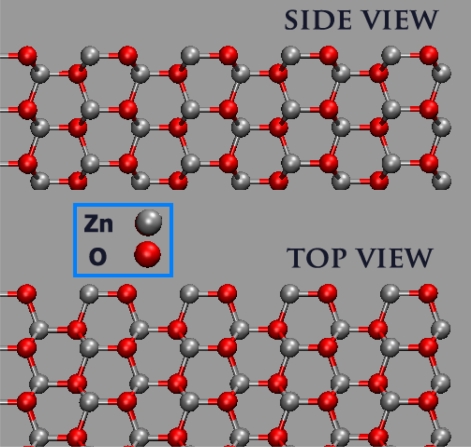
Side and top view of a clean and ideal ZnO (101^-^0) surface.

The nonpolar ZnO (101̄0) surface is electrostatically stable. This wurtzite–type surface consists of layers containing slightly tilted ZnO dimers, which are formed by three-fold coordinated Zn and O ions. These ZnO dimers assemble to form characteristic rows separated by trenches (see Figure 22). For the adsorption of water on this surface, one would expect that the oxygen atoms of the water molecules bind strongly to the coordinatively unsaturated Zn ions on the surface. This strong binding is due to a lock and key type interaction between the water molecules and the ZnO surface. The water molecules are stabilized by three different types of attractive interactions to both the substrate and neighboring adsorbate molecules. 1) The O atoms of the water molecules occupy the O sites of a hypothetical next ZnO layer on the ZnO (101̄0) surface so that the surface Zn ions regain their four-fold tetrahedral coordination as in the underlying bulk, which leads to a strong Zn-O and thus ZnO/H_2_O bonding. 2) One of the H atoms forms a hydrogen bond across the ZnO trench of the surface to a neighboring substrate O atom. 3) In the case of a convergent monolayer, a water-water hydrogen bond to a neighboring adwater is formed by the second H atom [82–84].

## 8. Conclusions and Perspectives

ZnO semiconductors have a range of unique properties, including their electronic properties and their size (in case of nanorods). The combination of these two important properties has driven extensive investigations in the last few years into their use in electrochemical devices, such as pH sensors. The application of ZnO nanorods as proton-sensitive electronic elements opens new challenges. In such applications, the size of the nanorods is the most important feature, not just in terms of their nanoscale diameters but also because their length is considerably greater than their diameter. This review highlights preliminary investigations of the ZnO/electrolyte interface. However, despite these gains, there are still tremendous opportunities and significant challenges to be solved. With regard to pH sensors, questions remain as to how commercializable devices can be made predictably and cheaply. The challenges of processing nanorods easily and integrating them with electronic devices in a simple manner are just beginning to be addressed. Using ZnO nanorods, it may be possible to make the ultimate nanoscale pH sensor, a single electronic element capable of single-ion recognition. Many of the challenges involving nanorods in bioelectronics also exist for applications where nanorods are to be integrated with living biological systems for use as intracellular pH sensors or ion-selective sensors.
